# Global issues, local action: exploring local governments use of research in “tackling climate change and its impacts on health” in Victoria, Australia

**DOI:** 10.1186/s12913-023-10087-5

**Published:** 2023-10-24

**Authors:** Jennifer Dam, Annemarie Wright, Joannette J (Annette) Bos, Peter Bragge

**Affiliations:** 1 Monash Sustainable Development Institute, Monash University, 8 Scenic Boulevard, Clayton Campus, Victoria, 3800 Australia; 2 Victorian Department of Health and Human Services, Victoria, Australia

**Keywords:** Local government, Knowledge Translation (KT), Research use, Public health, Normalization Process Theory (NPT)

## Abstract

**Background:**

Local government plays an important role in addressing complex public health challenges. While the use of research in this work is important, it is often poorly understood. This study aimed to build knowledge about *how* research is used by investigating its use by local government authorities (LGAs) in Victoria, Australia in responding to a new legislative requirement to prioritise climate and health in public health planning. The role of collaboration was also explored.

**Methods:**

Informed by Normalization Process Theory (NPT), this study adopted multiple research methods, combining data from an online survey and face-to-face interviews. Quantitative data were analysed using descriptive statistics; thematic analysis was used to analyse qualitative data.

**Results:**

Participants comprised 15 interviewees, and 46 survey respondents from 40 different LGAs. Research was most commonly accessed via evidence synthesis, and largely used to inform understanding about climate and health. When and how research was used was shaped by contextual factors including legislation, community values and practical limitations of how research needed to be communicated to decision-makers. Collaboration was more commonly associated with research access than use.

**Conclusions:**

Greater investment in the production and dissemination of localised research, that identifies local issues (e.g. climate risk factors) and is tailored to the communication needs of local audiences is needed to foster more impactful research use in local public health policy.

**Supplementary Information:**

The online version contains supplementary material available at 10.1186/s12913-023-10087-5.

## Introduction

### Global issues

The impacts of climate change on human health and wellbeing are a growing global concern; the effects of which are often experienced at the local level [1, 2]. In Australia and internationally climate-induced changes in weather events such as heatwaves, storms and floods, and associated impacts on air and water quality, food systems, spread of disease, and mental health stress are contributing to increased illness and mortality [1]. These impacts also interact with, and are frequently exacerbated by, social determinants of health such as age, gender, socio-economic status and public health infrastructure, often resulting in greater inequality in health outcomes [3].

The scale and urgency of this public health crisis requires action from all levels of government, including locally informed climate change adaptation and mitigation measures [4, 5]. Local governments, also known as municipalities, play a critical role in public health; responsible for improving the quality of life for their communities including social, economic and environmental viability and sustainability [6, 7]. Often described as the level of government closest to the people [7, 8], their connection to a defined population and place means they are well positioned to influence health outcomes both through both ‘bottom-up’ engagement with local stakeholders and ‘top-down’ policy interventions. Subsequently, climate change is part of an increasingly broad remit in the public health agenda for local governments in many regions [9–11].

In the international landscape, local government advocacy and engagement in climate action is well documented [3, 12]. For example, established networks such as the Global Covenant of Mayors for Climate and Energy [13], and Local Governments for Sustainability [14] work to foster coordinated action on sustainable urban development and building climate resilience at the local level. However, local governments face numerous challenges in developing local climate policy, including a lack of leadership or political will from higher levels of government, limited resources, institutional constraints, and availability and access to research [5, 9, 15, 16]. Overcoming these challenges and pursuing action to address the environmental and health impacts of climate change requires collaboration [2, 3, 10, 17]; both internally through cross-department engagement within individual local governments (e.g., health, environment and planning teams); and externally, through coordinated multi-sector action across all levels of government, the private sector, universities, advocacy groups and local communities [17].

The benefits of evidence-informed policy-making (i.e. the integration of contextual factors including community needs, political preferences and availability of resources alongside the best available research), are well documented [18–20]. In public health, the use of research is associated with improvements in health outcomes as well as service delivery and organisational efficiency [21]. Therefore, a compelling case exists to underpin climate health action with relevant research on both the nature and scale of climate-related health issues and the effectiveness of various interventions to address them [5, 15, 16].

After more than a decade of enquiry about research use in policy settings, the field of Knowledge Translation (KT) – the science of moving research into policy or practice [22] – has identified various factors that influence research use (e.g., staff skills, resources, time and organisational support [21]), however, it continues to grapple with identifying which strategies are expected to help optimise research use and why [23–26]. Building knowledge about *how* research is accessed and applied across different aspects of decision-making in policy settings, including local public health policy, is considered key to addressing this translational gap [27, 28].

### Local action: “tackling climate change and its impacts on health”

Over the past decade, pressure has grown in Australia for the adoption of a national framework to help drive coordinated action on climate and health [3, 4, 29]. State government action has been mixed however, the Victorian government (along with Queensland) was among the first to specify a health adaptation plan, and Victoria’s *Climate Change Act 2017* (Vic) identifies local government authorities (LGAs) as decision-makers that *must* consider climate change and its impacts on local communities.

Local governments’ public health responsibilities are also mandated through the *Public Health and Wellbeing Act 2008* (Vic), which requires local governments to develop a Municipal Public Health and Wellbeing Plan (MPHWP) every four years (s.26). While the Act specifies a “*principle of evidence based decision-making*” (s.5) to guide the effective use of resources and develop public health interventions, it also sets out requirements for collaborating with key stakeholders, community consultation, and examination of localised population health data in planning processes (s.26). It also stipulates that MPHWPs must have regard to the state public health and wellbeing plan [30].

In 2019, the Victorian government identified “*tackling climate change and its impacts on health*” as a priority area in the state public health and wellbeing plan, requiring local governments to adopt this new focus area in 2021–2025 MPHWPs [31]. Victorian local governments have been proactive in declaring a climate emergency and developing climate adaptation plans however, for many, this was the first time integrating a climate lens in public health planning. For MPHWPs to have the highest potential to protect and improve health and wellbeing, they need to be underpinned by research. To support this, the Victorian government issued a guidance document for local governments in 2020 to help inform “*scientific understanding of climate change and its impacts on health and councils’ experiences to date*” [31]. Given the mandates to draw on research, and the identification of climate change as a priority in planning, this represented an opportune moment to explore how Victorian local governments are mobilising to address the critical global issue of climate change and health, and more specifically, how research is being used to support this.

The purpose of this study was to provide a detailed exploration of how research is used in local government public health decision-making by examining how Victorian LGAs used research to support the integration of “*tackling climate change and its impacts on health*” in 2021–2025 MPHWPs. Specifically, this study aimed to examine:

- how research relating to climate change and health was accessed/sourced;
- when and how research was applied in public health planning and decision-making and;
- the role of collaboration and partnership in supporting research use in this context.

In doing so, this study hoped to provide valuable insights for researchers and policy-makers, locally and globally, that inform how to foster evidence-informed local government action on climate and health.

## Methods

### Study design

This study adopted multiple research methods, combining data from a state-wide online survey and interviews to support an in-depth exploration of how research was accessed and used to integrate climate change and health in 2021–2025 MPHWPs. Survey data was drawn from a related, but separate study that explored general aspects of research use and included specific questions about how research relating to climate and health was accessed, including use of the state government guidelines.

**Fig. 1:**
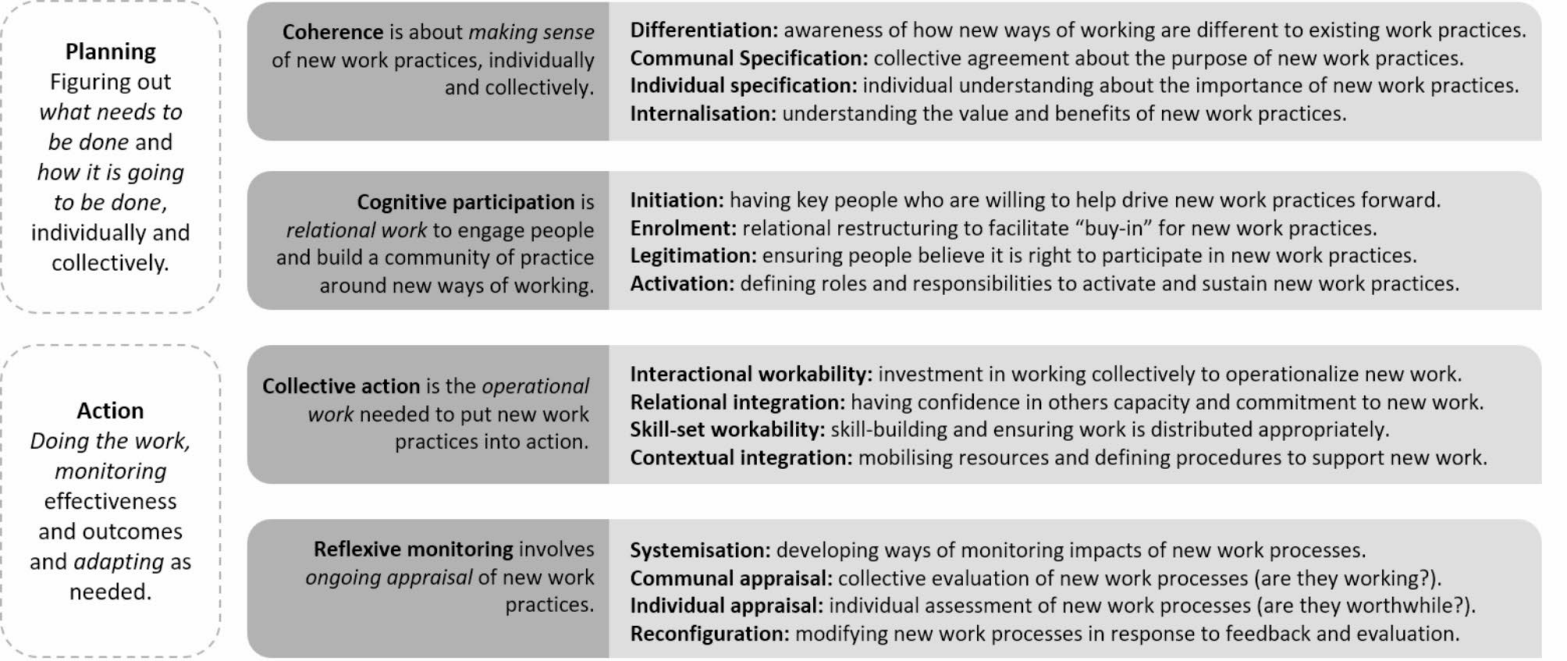
Normalization Process Theory implementation mechanisms Sources: [34–37]

The overall study design, including data collection and interpretation of results, was guided by Normalization Process Theory (NPT) [32, 33]. As an action theory, NPT proposes that the first step in understanding how a new practice becomes routine, or normalised, is to look at the work that people do both individually and collectively [32]. It focuses on the interactive and relational nature of implementation processes to help build understanding of how interventions or new ways of working are embedded and sustained in everyday work practices [33]. NPT comprises four mechanisms (or constructs), through which to understand factors that support or inhibit changes in practice (*coherence; cognitive participation; collective action; reflexive monitoring*) (see Fig. 1) [33–35]. NPT is an inherently fluid model, which encourages paying close attention to the iterative and dynamic nature of health improvement processes [34]. However, its use in qualitative research has shown that *coherence* and *cognitive participation* are often associated with planning and early stages of implementation projects due to their focus on making sense of new work processes and figuring out how they will be enacted; while *collective action* and *reflexive monitoring* are more often associated with experiences of enacting and evaluating new interventions or ways of working [36, 37].

### Sampling and participants

Also referred to as councils or municipal governments, there are 79 LGAs in Victoria, including 31 metropolitan and 48 rural or regional areas. Representatives from public health and sustainability/environment/climate teams (referred to henceforth as either ‘heath’ or ‘sustainability’ teams) from all 79 LGAs were eligible to participate in this study, providing they had contributed to the development of 2021–2025 MPHWPs.

*Interviews* were conducted with 15 representatives from 12 different LGAs, exploring how research is used to inform public health planning and strategy development to address climate and health. Of 55 *survey respondents*, 46 (across 34 different LGAs) responded to questions about accessing research relating to climate change and health, and are included in this study. Both interviewees and survey respondents had varying degrees of responsibility in their role/team (i.e. officers, planners, team leads), and a diversity of experience working in local government. Individual and LGA details for all participants are summarised in Table 1 below.

**Table 1:** Participant details

	Interviewees n = 15	Survey respondents n = 46	Total n = 61
**Work focus**			
Health	10	40	50
Sustainability	5	1	6
Other	0	5	5
**Work role**			
Manager/Lead	6	18	24
Planner/Officer	9	28	37
**LGA type**			
Regional/Rural	4*	22*	22**
Metropolitan	8*	12*	18**
Not stated	0	2	2

*Represents number of included LGAs from each sample, not number of participants

**Represents total number of included LGAs across both samples

### Procedure

Ethics approval to undertake this research was granted by the Monash University Human Research Ethics Committee (Project ID: 21,932). This research was undertaken in collaboration with the Victorian Health Promotion Foundation (VicHealth). VicHealth work across all levels of government and other sectors to develop and fund interventions, public health campaigns and research to promote public health outcomes [38]. VicHealth aided in developing and refining survey and interview schedules (helping to inform an understanding of planning processes), determining the timeframe for survey distribution and conducting interviews (i.e. being sensitive to planning cycles), and with participant recruitment.

### Online survey

Participants were recruited with the support of VicHealth and the Municipal Association of Victoria (MAV). Two emails were sent to a central contact at all 79 LGAs (approximately three weeks apart) by VicHealth’s Executive Lead, Policy and Research informing study details, a copy of the explanatory statement (Supplementary file 1) and an anonymous link to the online survey. Email recipients were asked to share the study details with all relevant staff. The study was also shared on a digital noticeboard for public health planners via the MAV, and on social media (i.e. LinkedIn) by the primary researcher.

Data collection took place from November 2021 to February 2022 using the online survey platform Qualtrics XM (2020). Prior to commencing the online survey, participants were asked to read the explanatory statement and record their informed consent. The online survey explored how research is used in local government public health decision-making (results reported elsewhere). A small subset of questions (see Supplementary file 2) asked about how research relating to climate and health was accessed, these results (which have not been previously published) are reported here.

### Interviews

In addition to support from VicHealth (i.e. sharing study details with regional planner networks via email), interview participants were recruited via a link at the completion of the online survey, on social media (i.e. LinkedIn and Twitter), and snowballing. All recruitment methods directed participants to register their interest via a Google form. Following this, an email was sent from the primary researcher providing a copy of the explanatory statement (see Supplementary file 3). Before being interviewed, those who wished to participate were asked to ensure they had read the explanatory statement and provide their written informed consent. Data collection took place from November 2021 to May 2022. Interviews were conducted by one researcher (JD) and were face-to-face (either online or in person), and audio recorded. To minimise burden on LGAs, the option was given to have multiple participants in one interview when more than one person expressed interest in taking part. One interview included two participants from a single LGA, all others were one-to-one.

Interviews were semi-structured to allow flexibility and sensitivity to differences in work processes between health and sustainability teams (see Supplementary file 4 for interview schedule). Participants were encouraged to draw on their experiences generally working in local government (both in current and previous roles), and more specifically about their involvement in the 2021–2025 planning cycle in which the new requirement to address climate and health was adopted.

Informed by NPT, interview questions focused on drawing out process-orientated descriptions of how research is accessed and used (including how it is communicated to various stakeholders), along with experiences of collaboration and partnership. Interview questions were piloted to ensure clarity and flow which resulted in some minor changes. Two additional changes were adopted after initial interviews. First, in setting the context, interviewees were asked to focus on the “*planning decisions*” phase of the planning cycle, which, as set out by the Victorian government requires the use of research [39]. However, as initial interviewees were either unfamiliar with the planning cycle or did not feel that it was reflective of their internal process (also noting that each phase is rarely done in isolation), a more holistic focus on “MPHWP development” was adopted to outline the interview context for the remaining interviews. Second, a modified interview schedule was developed for sustainability teams (removing the first block of questions relating to general use of research in public health planning as they were not relevant to this group), and introducing a question to elicit understanding of the connection between sustainability actions and health outcomes.

### Data analysis

Quantitative data were descriptively analysed using IBM SPSS Statistics (Version 28). Qualitative data was transcribed using a transcription service and analysed using NVIVO software (re-release 1.3). Thematic analysis (TA) was used to interpret and understand qualitative findings [40]. TA is considered a useful approach for qualitative research in health policy and practice, supporting robust data analysis and presentation of findings in accessible and meaningful ways [41]. Analysis involved several steps: transcripts were read through to become familiarised with the data and identify common or recurring ideas. Following this, initial codes were generated inductively to explore conceptual ideas and experiences of research use; and deductively to categorise process-orientated descriptions. Themes were constructed by organising codes into meaningful categories (refer Table 2 for overview). This was an iterative process, employing mind mapping along with NVIVO coding to explore relationships between codes, to allow for flexibility in theme development. Although this study was explicitly interested in research use, there were invariably accounts of the use of other forms of evidence. As researchers invested in optimising research use in public health decision-making, it was important to remain open to the importance of other forms of evidence in decision-making processes, including when and why it was valued. Five themes were constructed and defined. Results relating to how research was accessed and key themes are described, followed by a discussion of key insights learned using NPT as an interpretive lens.

**Table 2:** Alignment of research aims and themes

Research question	Themes
How is research accessed	Horizon scanning and deep diving: balancing depth of knowledge across a wide breadth of issues
When and how is research applied	Tackling the issue(s): climate change and its impact on health Legislation, community consultation, data and research: evidence needs and the priority setting context Measuring the immeasurable: the direct and indirect influence of research on policy decisions
What is the role of collaboration and partnership in accessing and applying research	Networks, partnerships, alliances, workshops, forums and working groups: the many forms of collaboration

## Results

### Accessing research

#### Survey respondents

Use of the state government’s guidelines was high (n = 44) amongst survey respondents. One respondent was not sure if they had been used, and one indicated that they were not needed as considerable planning work had already been undertaken before their release. Respondents were also asked to indicate how research relating to climate change and health was accessed more broadly, results are shown in Table 3.

**Table 3:** How survey respondents accessed research

	N = 46	
Source	Yes	No
Directly from academic journals including primary studies and systematic reviews	15 (32.6%)	31 (67.4%)
Professional networks, conversations with experts including academics, colleagues and practitioners	35 (76.1%)	11 (23.9%)
Synthesised sources such as evidence-based guidelines and reports, and research summaries	42 (91.3%)	4 (8.7%)

### Interviewees

Use of the guidelines was also high (n = 9) amongst interviewees, with many noting that they were an integral resource, providing synthesis of research evidence and informing understanding of the links between climate and health. One participant reported that the guidelines were not used in their LGA, a further two were unsure. Three reported limited use. Of these, two (from the same LGA) reported that research use overall was limited due to low levels of acceptance of climate science within their LGA; the third found the guidelines unhelp due to a lack of indicators and concrete actions.

Interviewees also emphasised the importance of research synthesis. The state government (and associated agencies) were the most commonly identified source of research synthesis, and this was often packaged with practical information for local governments to inform how to integrate climate change in health planning. Localised greenhouse gas alliances such as the South East Councils Climate Change Alliance (SECCCA) [42] were also important avenues for accessing and sharing research.

Sustainability teams reported more engagement with environmentally focused organisations, including Sustainability Victoria, CSIRO, the Intergovernmental Panel on Climate Change (IPCC), and the Department of Environment, Land, Water and Planning (DELWP). In contrast, health teams tended to rely more on peak health bodies such as VicHealth, the Cancer Council, and primary care partners or primary health networks. Peak bodies were widely considered a trusted source of research and a useful starting point for searching for research on a given topic:

*“VicHealth’s a good one, what programs they’ve done in the past and they sometimes have reports on, their links to previous studies or academic research and things like that.” (004, Health Team)*.

### Horizon scanning and deep diving: balancing depth of knowledge across a wide breadth of issues

Interviewees were in broad agreement about the importance of using research, however many reflected on the challenge of balancing the need to be informed about a wide range of issues, with having the capacity to dive deeper into the literature to tackle distinct priorities. ‘*Horizon scanning’* was a common practice:

*“…it’s certainly an ongoing process. It’s not something that’s only done in the sort of 6 months, 12 months leading up to the new public health and wellbeing plan… I’ve kind of built it into my almost daily routine that I sort of horizon scan what’s going on out there in the world in terms of updates, information, any research or anything like that across the whole spectrum of various health topics. So it’s something that I just constantly do, but I actually am not completely certain how valued or if my coordinator or my manager knew how much time I spent doing that, whether they would actually approve or not.” (007, Health)*.

While these processes were widely practiced and highly valued, allowing staff to build and maintain internal evidence repositories to draw on as needed in planning and decision-making processes, capacity to engage was often impacted by time and resource constraints (including dedicated research staff). This was particularly well articulated by one interviewee who had worked across two different LGAs:

*“here in XXX, we’d probably hire a consultant to do desktop research on all of the latest things, and give us the technical report and give us a whole lot of stuff that we could then draw on to develop. Whereas XXX just doesn’t have the money or the time to buy their expertise in and rely more on what’s more easily accessible.” (010, Sustainability)*.

For those with fewer resources, research summaries informing the nature and scale of different issues, and how to address them were highly valued:

*“in addition to data that said we have a problem that everyone could relate to, is the state government produced some really simple resources that took the evidence and translated them into relatable resources. And then we were all able to implement that.” (011, Health)*.

*“Sometimes it’s like, can you just give me the top three best buys. If we know we want to do this piece of work, what are the three must-haves?” (007, Health)*.

Interviewees also identified gaps in the availability of certain types of research. For example, localised research (particularly in regional LGAs) that was considered more influential with decision-makers; and demographically segmented research, that focused on the unique vulnerabilities of specific groups such as young people or the elderly in relation to climate change.

### Applying research

How and when research was applied was shaped by several contextual factors, including attitudes towards climate science, legislative requirements, evidence preferences of decision-makers and practicalities such as council resources and research availability.

#### Tackling the issue(s): climate change and its impact on health

A common reflection among interviewees was how the complexity of different public health issues (e.g., smoking; physical activity; climate change), and the degree of stakeholder acceptance shaped *how* and *when* research was used. When issues were well accepted, participants reported having more time to explore which actions to take, as opposed to ‘*making a case’* for action. In the case of climate change, which continues to be a contested issue at all levels of government in Australia, participants needed to invest time and resources in advocacy work and education to inform understanding and facilitate buy-in. As such, building knowledge and understanding of community values was considered equally important to building knowledge about the nature and scale of the issue.

*“So heat wave is a brilliant example of an opportunity to engage because the barriers to participation are typically low, the benefit is everyone knows someone who is at risk, and the things you do to make a substantial difference aren’t very painful for you… By contrast to urban heat island, intervening there, we might be talking about some changes to your lifestyle…. So the messaging is much more complicated and the barriers to people’s engaging in participating or accepting are much higher. (011, Health)*

Unique challenges associated with perceptions of the urgency of climate change were also described; and the willingness of councils to invest in actions associated with future (rather than immediate) gains. Addressing decision-makers’ desire to deliver tangible benefits to the community was an important factor shaping research needs:

*“…it can actually be a little bit tricky because a lot of places see that as such a long-term thing that they can’t influence it at the more immediate level.” (003, Health)*.

Building localised knowledge about specific issues affecting different communities (e.g., heat stress, bushfire, flooding, sea level rise, climate anxiety), and how to address them (e.g., asset management, awareness building, vulnerable person registers) were considered important steps in overcoming these challenges.

#### Legislation, community consultation, data and research: evidence needs and the priority setting context

For some LGAs incorporating climate change in public health planning was aligned with wider council priorities, however, in areas where acceptance of climate science was lower, it was a hurdle that was only made possible by the power of state-level legislation. Even when health issues were aligned with council agendas, legislation was a prevailing influence on decision-making, both in prioritising action on certain issues (i.e. climate change), and in prescribing research and wider evidence use (i.e. community consultation, population health data):

*“I think it does give a significant influence because in a Council report, up the top, you have to put whether it’s a legislative requirement and the context.” (004, Health)*.

The value of legislation was emphasised by this participant:

*“Now, we actually have a much stronger legislative framework with the local government in the Climate Change Act where we can actually say to our councillors, actually we have to do something about this. It’s required. It’s more about the scale of what you deliver, and the extent to which you deliver a program within that.” (010, Sustainability)*.

The diversity of local government stakeholders, including their values and communication and evidence preferences also needed to be considered:

*“…in local government, as a person who’s trying to implement some public health and wellbeing intervention, you’ve got two or three critical sets of stakeholders. So you’ve got the rest of your organisation, … the community at large and particularly the community that your service area is directly engaged with. And very importantly is councillors who have a view of what their community is, what it aspires to be, and how to close the gap.“ (011, Health)*.

*“At any council you can get councillors with such diverse range of knowledge, experience, skills, any given topic.” (004, Health)*.

Tailoring communication and reporting (and supporting evidence) according to the information needs of different stakeholders was common practice. For councillors, outputs from community consultation and data (particularly local data), were considered most influential and therefore, often more helpful than research in making a case for a particular course of action.

#### Measuring the immeasurable: the direct and indirect influence of research on policy decisions

While at times, the role of research appeared secondary to other forms of evidence, descriptions of its use (both directly and indirectly) suggest that research engagement is purposeful, sometimes strategic, and more pertinent in certain aspects of planning than others. For example, in highlighting how research can be an avenue to activate conversation about “*tricky*” issues, to inform and educate stakeholders (e.g., about the links between climate and health), and to identify the best course of action once priorities have been established:

*“I think the up-to-date information in terms of data, is often what is a key driver with a municipal public health and wellbeing plan. The actual use of the evidence, which is that sense of, okay, we know what the situation is because here’s the data, but what do we do about that is the point at which we need to connect the dots.” (001, Heath).*

Descriptions about the relative impact of research in decision-making processes centred around organisational appetite for research use, and the very practical limitations of the “*two-pager*”:

*“But once again, everything’s just heavily pared back. So it’s really challenging to pitch some of the scientific stuff. … If you’re presenting something to the executive, you spend time writing a paper and it’s got to be a two-page paper and you might actually have a draft strategy that’s 60 pages long, but this process that you’ve gone through, you’ve got to condense it down to two pages.” (011, Health)*.

When decision-maker’s (i.e. councillors and CEOs) appetite for research was high, interviewees spoke confidently about the direct influence of research in decision-making processes. However, many reported a *“gap”* between the knowledge that they gathered and *“being able to use that to influence the decisions”* (007, Health). While this was a source of frustration for some, most were pragmatic in their expectations about research influence. However, decisions about whether to communicate research in planning proposals varied:

*“I’m in a very lucky position because as a policy officer, my job is to seek the knowledge, consult with the community, and provide it up, whether they like it or not. But in the end, they make the decisions around what will be used, how much will be used, and what they will land with in terms of policy. But in my role, I will send it up the line. " (005, Health)*.

*“… it’s essential for us as policy as writers and policy recommenders that we understand the evidence, because there’s no point proposing a program if you just don’t have any sense it’s going to work. …But then how that evidence is used to convince the decision-makers to agree with the proposals depends on the audience.” (010, Sustainability)*.

### Collaboration

Working in partnership with the Department of Health and other agencies to accomplish public health goals is a legislative requirement for local governments [30]. Interviews explored how participants engaged in collaborative action, and how engagement supported (or inhibited) research use (along with other aspects of public health planning).

#### Networks, partnerships, alliances, workshops, forums and working groups: the many forms of collaboration

While there was no one-size-fits-all approach, interviewees were emphatic about the importance of collaboration in fostering broad stakeholder buy-in and promoting strategic and coordinated action in public health planning generally, and in the context of addressing climate and health:

*“I think that’s one of the key things about a municipal public health and wellbeing plan, it cannot be led by one individual. It needs to be led and owned by a number of people to make it really impactful.” (001, Health)*.

Direct engagement with peers and experts for the purpose of accessing research was most common among participants who had previous experience working in research settings (e.g. university academics). However, a shared investment in evidence-informed decision-making (e.g. across state organisations, peak bodies and greenhouse gas alliances) meant that collaboration frequently served dual benefits of capacity building, as well as being important avenues for accessing research. For example, regional greenhouse gas alliances that connected neighbouring LGAs providing opportunities to pool resources and undertake shared projects offered numerous benefits:

*“Well, firstly, we get someone to run a project that we don’t have to do ourselves. And we get the benefit of all of the different inputs from all of the different councils into we get the benefit of having that collective minds thinking about it. You get consistency.” (008, Sustainability)*.

Internal collaboration between health and substantiality teams was also discussed. Most interviewees reported some degree of engagement in exploring how to approach climate and health planning, often drawing on work that had been previously undertaken. Others embraced the opportunity to develop more structured approaches, and benefited from opportunities to develop actions that offer dual benefits for both environmental and health outcomes:

*“the delivery of the climate emergency action plan doesn’t sit with one person, it’s the whole of organization approach. And same with the health and wellbeing plan.” (004, Health)*.

*“… we have internal partners as well … We have to keep working with them so we have that relationship and as part of the planning” (003, Health)*.

*“…some of those health priorities will be addressed through looking for co-benefits, looking for existing work that’s going on at council and finding opportunities to address the health priorities within the existing work…” (009, Health)*.

Two interviewees were unaware of whether opportunities exist for collaborating with local partners about climate and health, suggesting that internal collaboration is also important for connecting health teams with wider networks:

*“The sustainability environmental stuff is not my area of expertise, so I don’t personally have strong networks in that space… that doesn’t mean that there aren’t people in the organization who might not have those networks established… that may reflect my expertise and my linkages and networking, rather than what’s happening on the ground.” (001, Health)*.

### Understanding research use through the lens of NPT

#### *Sense-making work*: using research to understand climate and health, and figure out what needs to be done

*Sense-making* requires attention to both individual and shared understanding of new work practices and why they matter. Although climate action is not a new remit for Victorian local governments, prior research and planning have predominantly centred on environmental outcomes such as reducing greenhouse gas emissions and addressing biodiversity loss [12]. While participants were quick to acknowledge the natural synergies between improvements in environmental outcomes and positive impacts on health, both health and sustainability teams identified the importance of building their own and others’ understanding of the links between climate and health. Research was widely considered to be a critical resource in building this understanding; both to inform planning and strategy development, and for equipping teams to build awareness amongst councillors and communities.

Although identifying local risk factors and strategies to address them was articulated as an important aspect of planning processes, using research to inform this work presented a number of challenges. Participants lamented a lack of locally informed research about identifying climate risk factors to directly address the evidence needs of local decision-makers. While some LGAs had resources to invest in commissioning local research, many did not. Similarly, capacity to engage with research-based organisations (e.g., universities) to help build local knowledge, was also hindered by a lack of resources.

#### *Relational work*: fostering research engagement through reframing and building communities of practice

*Relational* work involves fostering stakeholder buy-in and developing a plan for action to help drive new practices forward [32]. As a key focus of this study, participants described myriad ways they engage with peers and wider networks to build and share knowledge. They were also cognisant of the importance of building and maintaining relationships with councillors and community to be effective in their respective roles, including knowing when and how to best communicate research. As a politically contested issue in many LGAs, talking about the impacts of climate on health needed to be navigated sensitively, and often without directly referring to climate science. Some participants reported engaging in strategies such as ‘reframing’ [9] to present information in more socially acceptable ways. For example; emphasising the cost benefits of pro-environmental actions (e.g., solar panels), caring for the wellbeing of loved ones in extreme heat, or emergency planning for flooding events; were all easier conversations to have, provided issues had local salience.

Engaging in communities of practice such as alliances and professional networks are also considered important aspects of r*elational work* [35]. According to NPT this type of collective engagement can help energise new practices and drive them forward [43]. As well as providing opportunities for coordinating resources and sharing ideas to inform MPHWP development, collaboration with peers and professional networks was also articulated by interviewees as an important driver of research use by facilitating awareness of and access to relevant research.

## Discussion

Victorian local governments’ public health responsibilities are governed by the *Public Health and Wellbeing Act 2008* (Vic) which specifies requirements for using research and the adoption of state government public health priority areas; informing the recent inclusion of “*tackling climate change and its impacts on health*” 2021–2025 MPHWPs [31]. This study examined research use in addressing this new priority. Findings identify the importance of evidence synthesis for accessing research. Exploration of *how* research was applied highlighted both direct and indirect research use in public health policy, and that *how* it is used is shaped by wider factors such as the nature of the issue being addressed (i.e. climate change and health), state government legislation and community values. Collaboration, in all its forms, was an important facilitator of research use.

### Accessing research

This study found that evidence synthesis (e.g., guidelines, research summaries) and professional networks were important avenues for accessing research; direct engagement with primary research was less common. These findings are consistent with wider literature [20, 44], which underscores the importance of high-quality, up-to-date synthesis to support research use in local policy settings [45, 46]. Previous research within Victorian LGAs highlights the impact of access to academic databases and confidence (i.e. in assessing research quality or trustworthiness) on research use [47]. While these factors surfaced in the present study, participants also emphasised the challenge of staying ‘up-to-date’ with research across a broad range of health issues. Evidence synthesis was therefore critical to addressing time and resource constraints. Additionally, state government departments and peak bodies were perceived by many participants as being trustworthy sources in the eyes of decision-makers and therefore more likely to be accepted. While there was some overlap in how research was accessed between public health and sustainability teams, considerable diversity was observed in terms of how information was made available by the state government (e.g. workshops, forums, and guideline documents), emphasising the importance of sharing research in a variety of ways to meet the diverse needs of local government stakeholders.

### Applying research

This study drew on NPT as an interpretive lens to inform a deeper understanding of the practicalities of research use. According to NPT, implementation of new interventions or ways of working is achieved through a series of mechanisms (i.e. *coherence, cognitive participation, collective action* and *reflexive monitoring*; see Fig. 1) “*that motivate and shape the work that people do when they participate in implementation processes*” [32]. Data analysis in this study drew on two mechanisms: *coherence* (also referred to as *sense-making)*, which involves building understanding about new practices, why they matter, and what needs to be done and; *cognitive participation* (or *relational* work) which involves fostering buy-in and figuring out *how* this work will be done [35]. These processes have been identified in previous studies as important factors in the successful implementation of new interventions [48, 49].

Research was an important tool in *sense-making* work, helping participants build their understanding of the links between climate and health, and confidence to educate wider stakeholders (e.g. councillors and communities). This work required a significant investment from public health teams, likely due to a widespread lack of awareness about the health impacts of climate amongst local communities in Victoria [50]. At times this impacted the degree to which teams were able to invest in the challenge of identifying what work needs to be done, this was also impacted by a paucity of research describing localised risk factors and how to tackle them [3, 10, 11, 51, 52].

Added to this challenge, the politically contested nature of climate science in some LGAs meant that alongside building understanding, public health teams also needed to invest in *relational* work to foster buy-in with communities and councillors. An important aspect of relational work is *legitimation*, or the belief that prioritising climate change in public health planning is right [35]. While legislation was widely identified as a key driver for the prioritisation of climate and health in MPHWPs, it was much less sufficient in shifting beliefs, making this an important task for public health teams. Although this work was approached in different ways (e.g. ‘reframing’ narratives in socially acceptable ways or emphasising the economic impacts of climate change); participants commonly highlighted the importance of having a clear understanding of the needs and values of key stakeholders. While participants demonstrated considerable flexibility in their use of research in responding to these diverse needs and values, this finding highlights the importance of building capacity for research communication to support wider research engagement and promote action on climate and health [51].

Taken together, these findings add to wider calls in Australia and internationally for greater investment in research to inform policy responses to climate and health [3, 10, 11, 51, 52], including research that is informed by local communities and supports values-based messaging by addressing the diverse information needs of stakeholders in local government settings [9].

### Collaboration

Collaboration is an important facilitator of research use [53–55], and critical to addressing the often complex challenges encountered in public health [44, 56], including climate change [17, 57]. While the importance of collaboration was evident in this study, it was more often associated with research access than use. Although reported engagement in shared projects to commission research or implement evidence-based strategies were less common, cross-council collaboration was considered important offering benefits of collective investment and thinking and promoting a consistent approach. This highlights an opportunity for greater investment in cross-council engagement, for example in regions that share common climate risk factors, to develop common strategies to address them.

Collaboration was important to both public health and sustainability teams; however, differences were observed (i.e. health teams were more reliant on traditional health partners such as primary care partners and health promotion organisations while sustainability teams had more climate-focused networks). Furthermore, this study observed a positive association between internal collaboration (i.e. between health and sustainability teams within a single LGA), and increased awareness of climate-focused organisations for public health teams. LGAs that reported consistent approaches to internal collaboration described numerous benefits, including knowledge and resource sharing, identification of actions that offer co-benefits for health and the environment, and greater networking opportunities to support research engagement and use. By fostering collaboration amongst internal stakeholders, LGAs can help facilitate the *relational work* needed to support the adoption of new work practices, and promote wider research engagement in these processes [35].

#### Implications for research and policy

Findings from this study present several practical and theoretical implications for research and policy. In focusing on the issue of climate change and health, this study also highlights a need in local government settings for plain language research (including research synthesis) that communicates the links between climate and health, and more localised research that informs the type of risk factors impacting health outcomes in local communities, and how to address them. For example, case studies that describe how local governments apply research to undertake climate risk assessment and how associated interventions are practically contextualised and implemented in local settings. In the absence of locally informed research, investment in capacity building to support the translation of global research into local contexts may also be of value [11].

For example, there is a growing body of national and international literature that highlights the leading role of local governments in climate change innovation [58], and the dual benefits that can be achieved for health and the environment through climate friendly planning and policy development [58–60]. Urban greening strategies is one such example which offers clear benefits for addressing the symptoms and risk factors of numerous chronic diseases (i.e. mental health disorders, obesity and cardiovascular disease) [61, 62], as well as helping to address the health and environmental impacts of climate change [63] through improved air quality, cooling and shade, and reduced risk of flooding. This literature offers a rich resource for local governments to draw on to better understand climate related health issues, alongside evidence-based actions to address them.

By exploring research use in a particular aspect of decision-making (i.e. planning and strategy development) and on a particular issue (climate and health), this study builds on previous research demonstrating that viewing research use through the lens of policy-making processes can help identify how and why research is used. Consistent with Mackenzie et al. [48], this study found that the use of NPT helped bring attention to the interactions between various actors and processes in local public health planning; shedding light on how research is used and providing insight into the information needs of different stakeholders. While findings highlight opportunities to build individual capacity for mobilising research, the identification of institutional constraints, including those arising from internal organisational structures and those resulting from higher levels of government (e.g. legislation) underscore a need for systems level change. This includes stronger leadership at all levels of government on climate change, as an issue of significant local and global concern [5, 58]. If local government decision-makers are to meet growing demands for greater action in tackling climate change and health, there needs to be greater investment in building individual and organisational capability to do so.

### Strengths and limitations

A key strength of this study is the use of multiple methods and rigorous research methodology in data collection and analysis. Flexible use of thematic analysis in coding interview data (including both inductive and deductive analysis) supported the construction of themes that captured both process-orientated descriptions of research use as well as more conceptual ideas relating to when, how and why research is applied (or not applied). The targeted focus on climate and health allowed this study to generate specific insights about research use in this context and more generalisable insights about how research needs may differ depending on the nature of the issue being addressed and the type of decisions being made. Finally, the use of NPT as an interpretive lens helped to garner further insights about research use in direct relationship to implementation processes. Furthermore, the methods used in this study offer a replicable approach to exploring research use in tackling climate and health in other jurisdictions and settings, which is an important secondary contribution of this paper.

There were also limitations. While the direct focus on planning and strategy development provided important insights into research use in this context, these insights may be less applicable to understanding research use in wider aspects of decision-making. Generalisability of findings to wider settings is further impacted by a relatively small sample size for both the survey and interviews. Additionally, although research methods and survey and interview schedules were developed in consultation with all four study authors, data collection and analysis were primarily undertaken by one researcher (JD). This is likely to have influenced data collection and analysis, subsequently, the researcher sought to engage reflexively, particularly in interviews, acknowledging their subjectivity in these processes. Despite a desire to focus exclusively on research use in interviews, for participants, it was difficult to separate research from other forms of evidence. This led the interviewer to adopt a wider viewpoint, engaging with why research was *not* used, as well as when and why it was. A final limitation of this study was that it did not include councillors or community groups. As key stakeholders in public health planning, their engagement has a clear impact on when and how research is used and as such a better understanding of their research needs, and perceptions of research may benefit KT researchers aiming to foster greater use of research in this sector.

## Conclusion

This study explored research use by Victorian local governments in implementing a new requirement in public health and wellbeing planning: *“tackling climate change and its impacts on health”.* Findings revealed that while research engagement amongst health and sustainability teams is high, the direct application of research in planning decisions can vary. Reasons for this include variations in the perceived evidence needs of different decision-makers, the degree of acceptance of climate science amongst stakeholders, and various practical limitations such as having to summarise evidence into a two-page document. By examining research use in the context of integrating a climate lens in health planning, this study observed that research use for this purpose was largely associated with the NPT concepts of *sense-making* (the work of building understanding about the links between climate change and health), and the *relational* work of building communities of practice. Collaboration in all of its forms was essential in negotiating how to address this new legislative requirement, and in associated research use.

Overall, this study found that fostering more impactful use of research in local public health policy would benefit from the production and dissemination of research that better engages with how local governments approach the work of public health (e.g. place-based, or demographically focused), addresses the challenges associated with different aspects of decision-making (e.g. public health planning), and is tailored to the communication needs of local audiences.

### Electronic supplementary material

Below is the link to the electronic supplementary material.

Supplementary Material 1

Supplementary Material 2

Supplementary Material 3

Supplementary Material 4

## Data Availability

Aggregated, anonymised data is available from the corresponding author on reasonable request.

## Abbreviations

KT: Knowledge Translation
LGA: Local Government Authority
MPHWP: Municipal Public Health and Wellbeing Plan
SECCA: South East Councils Climate Change Alliance
IPCC: Intergovernmental Panel on Climate Change
DEWLP: Departmental of Environment, Water, Land and Planning
NPT: Normalization Process Theory
