# Supplementary material for: Global issues, local action: exploring local governments use of research in “tackling climate change and its impacts on health” in Victoria, Australia
Source: BMC Health Serv Res. 2023 Oct 24;23:1142. doi: 10.1186/s12913-023-10087-5 (PMC10594743; doi:10.1186/s12913-023-10087-5)
Supplement: Supplementary file 1 — Supplementary Material 1 [file 12913_2023_10087_MOESM1_ESM.doc]

**EXPLANATORY STATEMENT**

**Survey Participants**

**Project ID:** 21932

**Project title:** Exploring the use of research evidence in Municipal Public Health and Wellbeing Planning in Victoria

| **Associate Professor Peter Bragge** Monash Sustainable Development Institute | **Dr Annemarie Wright**  VicHealth | **Ms Jennifer Dam** Monash Sustainable Development Institute |
| --- | --- | --- |

You are invited to take part in this study. Please read this Explanatory Statement in full before deciding whether or not to participate in this research. If you would like further information regarding any aspect of this project, you are encouraged to contact the researchers via the phone numbers or email addresses listed above.

**What does the research involve?**

This study is part of a doctoral research project aimed at exploring how research evidence is used in local government public health and wellbeing planning and identifying ways to foster greater use of research evidence.

Participation in this study involves completing a brief online survey. The survey will take approximately 10-15 minutes to complete and can be done at a time that best suits you.

The survey will ask questions about your experiences of using research evidence in municipal public health and wellbeing planning including:

- The types of research evidence used and where it is sourced from
- How research evidence is used at different stages of the planning cycle
- How the use of research evidence is integrated into everyday work practices
- Research evidence use to address the new Victorian Government focus area “tackling climate change and its impact on health”

**Why were you chosen for this research?**

Staff or consultants from all local governments in Victoria, Australia who are involved in municipal public health and wellbeing planning are invited to participate in this study.

**Source of funding**

This study forms part of a broader PhD research project within the Behaviour Change Graduate Research Industry Partnership program within the Monash Sustainable Development Institute, at Monash University. The student (Jennifer Dam) and project are funded by the Victorian Health Promotion Foundation and the Australian Government Research Training Program (RTP) Scholarship.

**Consenting to participate in the project and withdrawing from the research**

Participation in this study is entirely voluntary. By completing the online survey and submitting your responses you are consenting to participate in this study. You can choose to withdraw from the study at any time by exiting the survey before hitting submit. However due to the survey being anonymous withdrawal will not be possible once you have submitted your responses. Data will be de-identified and reported in aggregate in research outputs (journal articles, conference presentations and the final PhD thesis). Data will be de-identified and reported in aggregate in research outputs including journal articles, conference presentations and the final PhD thesis.

You will be provided with the opportunity to provide your contact details at the end of the survey if you are happy to be contacted in relation to this or future research projects. By providing your details you are not consenting to participating in any future studies and you will have the opportunity to decline participation if you are contacted by the research team at another point in time.

**Possible benefits and risks to participants**

This study aims to capture the practical experiences of local governments in using research evidence at different stages of the planning cycle to provide much needed evidence on *how* local governments are using this type of evidence in public health and wellbeing planning. It will provide important insights for local governments across Victoria and globally on how the work of using research evidence in public health and wellbeing planning is embedded in everyday practices.

There is little risk associated with participating in this study. The survey can be completed at a time and location of convenience to the participant. Data will be de-identified and reported in aggregate. The nature of the study and the data being collected is not sensitive however, survey respondents will have the option of withdrawing from the study at any time by exiting the survey before hitting submit.

**Confidentiality**

The survey will be completely anonymous and data will be reported and analysed in aggregate to ensure neither organisations or individual participants are identifiable. While participants will not be asked to identify themselves in completing the survey, they will be invited to provide their contact details if they are happy to be contacted about participating in future research. Contact details will not be linked to survey responses which will be kept anonymous. They will be stored separately from the survey response data, all of which will be stored electronically only and kept in the secure Monash google and network drives.

**Storage of data**

The survey will be delivered via the Qualtrics online platform, which is supported by Monash University. Files will be transferred to a laptop running on the Monash standard operating environment and stored in a google shared drive with the student (Jennifer Dam) and her supervision team (A/Prof Peter Bragge and A/Prof Annette Bos, Monash University, and Dr Annemarie Wright, VicHealth). A back-up copy of files will be saved on the secured Monash network drive. These data storage options have been chosen in line with advice provided by the Monash Research Office. All files, electronic and paper-based will be destroyed securely after five years, as per legislative requirements.

**Use of data for other purposes**

Raw data from this study will only be made available on request and will be fully anonymised. Data may be requested for use in other projects however, only aggregate de-identified data may be used and only where ethics approval has been granted.

**Results**

Research outputs from this study may include peer-reviewed publication/s, inclusion in the student’s final thesis as part of her doctoral degree, presentation at academic and/or industry conferences and articles in non-academic publications such as The Conversation. Request for copies of any research outputs can be made to the student investigator ([jennifer.dam@monash.edu](mailto:jennifer.dam@monash.edu))

**Complaints**

Should you have any concerns or complaints about the conduct of the project, you are welcome to contact the Executive Officer, Monash University Human Research Ethics Committee (MUHREC):

| Executive Officer Monash University Human Research Ethics Committee (MUHREC)  Room 111, Chancellery Building D, 26 Sports Walk, Clayton Campus Research Office Monash University VIC 3800  Tel: +61 3 9905 2052 Email: [muhrec@monash.edu](mailto:muhrec@monash.edu) Fax: +61 3 9905 3831 |  |
| --- | --- |

Thank you,
**Peter Bragge**
