# Supplementary material for: Global issues, local action: exploring local governments use of research in “tackling climate change and its impacts on health” in Victoria, Australia
Source: BMC Health Serv Res. 2023 Oct 24;23:1142. doi: 10.1186/s12913-023-10087-5 (PMC10594743; doi:10.1186/s12913-023-10087-5)
Supplement: Supplementary file 2 — Supplementary Material 2 [file 12913_2023_10087_MOESM2_ESM.docx]

**SUPPLEMENTARY FILE 2**

**Survey Questions (Sections 2 and 5, as used in this study)**

Welcome to the research study! We are interested in understanding how local governments use research evidence in public health and wellbeing planning. This survey should take around 10-15 minutes to complete. Your responses will be anonymous, participation is voluntary, and you have the right to withdraw at any point during the study. Before continuing, please ensure you have read the [Explanatory Statement](https://monash.az1.qualtrics.com/WRQualtricsControlPanel/File.php?F=F_7aK9k2gJfzF0w8S). The Principal Investigator of this study is XXX, he can be contacted at: XXX

By clicking yes below, you acknowledge:

1. Your participation in the study is voluntary

2. You are 18 years of age or older

3. You are aware that you may choose to terminate your participation at any time for any reason

- I consent, begin the study
- I do not consent, I do not wish to participate

In the space below, please advise your current position. Please answer all the survey questions from the perspective of this position.

*Section 2: About you*

What is your age group?

- 18-29
- 30-39
- 40-49
- 50-59
- 60+

Which local government do you currently work for?

Are you a consultant or directly employed?

- Employee
- Consultant

How many years have you worked with/within local government?

- Less than 1 year
- 1-3 years
- 4-6 years
- 7-9 years
- 10 or more years

How many years have you worked in your current position?

- Less than 1 year
- 1-3 years
- 4-6 years
- 7-9 years
- 10 or more years

What is your highest level of qualification?

- High School
- Certificate
- Diploma/Advanced Diploma
- Bachelor Degree (inc. Honours)
- Graduate Diploma/Graduate Certificate
- Post Graduate Degree (Masters)
- Post Graduate Degree (PhD)

What is your field of expertise?

*Section 5: Using evidence to tackle the impacts of climate change on health*

"Tackling climate change and its impacts on health" is a new focus area that local governments in Victoria are required to address through municipal public health and wellbeing planning. Did your organisation incorporate "tackling climate change and its impacts on health" in its Municipal Public Health and Wellbeing Plan 2021-2025?

- Yes
- No

To support local governments in integrating this new focus area in MPHWPs, the Department of Health and Human Services developed guidelines titled "Tackling climate change and its impacts on health through municipal public health and wellbeing planning: Guidance for local government, 2020". Did you/your organisation use these guidelines when developing your MPHWP 2021- 2025?

- Yes
- No

Where else did you source research evidence to address incorporating "tackling climate change and its impacts on health" in your public health and wellbeing plan? (Please select all that apply)

- Through my professional networks and conversations with experts such as academics, colleagues and practitioners
- Through synthesised sources such as guidelines and reports for example state/national evidence-based guidelines or research summaries
- Directly from academic journals including primary research studies and systematic reviews
