# Supplementary material for: Global issues, local action: exploring local governments use of research in “tackling climate change and its impacts on health” in Victoria, Australia
Source: BMC Health Serv Res. 2023 Oct 24;23:1142. doi: 10.1186/s12913-023-10087-5 (PMC10594743; doi:10.1186/s12913-023-10087-5)
Supplement: Supplementary file 3 — Supplementary Material 3 [file 12913_2023_10087_MOESM3_ESM.doc]

**EXPLANATORY STATEMENT**

**Interviewees**

**Project ID:** 21932

**Project title:** Exploring the use of research evidence in Municipal Public Health and Wellbeing Planning in Victoria

| **Associate Professor Peter Bragge** Monash Sustainable Development Institute | **Dr Annemarie Wright**  VicHealth | **Ms Jennifer Dam** Monash Sustainable Development Institute |
| --- | --- | --- |

You are invited to take part in this study. Please read this Explanatory Statement in full before deciding whether or not to participate in this research. If you would like further information regarding any aspect of this project, you are encouraged to contact the researchers via the phone numbers or email addresses listed above.

**What does the research involve?**

This study is part of a doctoral research project aimed at exploring how research evidence is used in local government public health and wellbeing planning and identifying ways to foster greater use of research evidence.

Participation in this study involves being interviewed. Interviews are expected to take approximately 60 minutes and can be done at a time that best suits you. Given the ongoing uncertainty surrounding COVID19 restrictions, it is likely that interviews will be conducted via zoom however, if restrictions allow, and it is preferable, arrangements may be made to conduct interviews face to face. Interviews may be audio recorded.

The interview will ask questions about your experiences of using research evidence in municipal public health and wellbeing planning with a focus on:

- Collaboration and partnership activities that support research evidence use
- Research evidence use to address the new Victorian Government focus area “tackling climate change and its impact on health”

**Why were you chosen for this research?**

Staff or consultants from all local governments in Victoria, Australia who are involved in municipal public health and wellbeing planning are invited to participate in this study. This may include staff from other departments (e.g. whose main focus is sustainability or climate change) but whose expertise has been engaged in public health and wellbeing planning.

**Source of funding**

This study forms part of a broader PhD research project within the Behaviour Change Graduate Research Industry Partnership program within the Monash Sustainable Development Institute, at Monash University. The student (Jennifer Dam) and project are funded by the Victorian Health Promotion Foundation and the Australian Government Research Training Program (RTP) Scholarship.

**Consenting to participate in the project and withdrawing from the research**

Participation in this study is entirely voluntary. A consent form will be provided to you prior to the interview for your review and signature and a copy can be provided to you on request. You can choose to withdraw from the study up to 48 hours after the interview has been conducted by contacting the interviewer in writing (via email). Data will be de-identified and reported in aggregate in research outputs (journal articles, conference presentations and the final PhD thesis).

**Possible benefits and risks to participants**

This study aims to capture the practical experiences of local governments in using research evidence in public health and wellbeing planning with a particular emphasis on how it is used to tackle climate change and its impacts on health, and on the role of collaboration and partnership. This research aims to provide insights on local government experiences of using research evidence to tackle climate change and health, which is a significant risk factor in human health and wellbeing locally and globally.

There is little risk associated with participating in this study. Interviews will be conducted at a time and location of convenience to the participant. Data will be de-identified and reported in aggregate. The nature of the study and the data being collected is not sensitive however, you will have the option to pass on any questions you do not feel comfortable answering.

**Confidentiality**

Pseudonyms will be used for all participants in transcription, analysis and reporting of data. Participants will not be asked to identify themselves in the interview recordings, the researcher will keep records of interviews and pseudonyms, all of which will be stored electronically only and kept in the secure Monash google and network drives. If direct quotes are used in journal articles, in the final thesis or at a conference, only pseudonyms will be used. Interview recordings may be transcribed by an external party.

**Storage of data**

Interviews will be recorded via Zoom if conducted online or using a university owned handheld recording device if conducted in person. Files will be transferred to a laptop running on the Monash standard operating environment and stored in a google shared drive with the student (Jennifer Dam) and her supervision team (A/Prof Peter Bragge and A/Prof Annette Bos, Monash University, and Dr Annemarie Wright, VicHealth). Once transferred to the laptop, the files will be deleted from the handheld recording device. A back-up copy of files will be saved on the secured Monash network drive. These data storage options have been chosen in line with advice provided by the Monash Research Office. All files, electronic and paper-based will be destroyed securely after five years, as per legislative requirements.

**Results**

Research outputs from this study may include peer-reviewed publication/s, inclusion in the student’s final thesis as part of her doctoral degree, presentation at academic and/or industry conferences and articles in non-academic publications such as The Conversation. Request for copies of any research outputs can be made to the student investigator ([XXX](mailto:jennifer.dam@monash.edu))

**Complaints**

Should you have any concerns or complaints about the conduct of the project, you are welcome to contact the Executive Officer, Monash University Human Research Ethics Committee (MUHREC):

| Executive Officer Monash University Human Research Ethics Committee (MUHREC)  Room 111, Chancellery Building D, 26 Sports Walk, Clayton Campus Research Office Monash University VIC 3800  Tel: +61 3 9905 2052 Email: [muhrec@monash.edu](mailto:muhrec@monash.edu) Fax: +61 3 9905 3831 |  |
| --- | --- |

Thank you,
**Peter Bragge**
