# Supplementary material for: Global issues, local action: exploring local governments use of research in “tackling climate change and its impacts on health” in Victoria, Australia
Source: BMC Health Serv Res. 2023 Oct 24;23:1142. doi: 10.1186/s12913-023-10087-5 (PMC10594743; doi:10.1186/s12913-023-10087-5)
Supplement: Supplementary file 4 — Supplementary Material 4 [file 12913_2023_10087_MOESM4_ESM.docx]

**SUPPLEMENTARY FILE 4**

**Interview Schedule (semi-structured approach)**

Context: The Victorian Government outlines a six-stage planning cycle which recommends key actions and milestones that local governments should aim to achieve as part of the process of developing Municipal Health and Wellbeing Plans. This interview aims to explore the role of research evidence in “planning decisions” including how it is gathered, applied and communicated.

1. How is research evidence gathered to inform planning decisions?
   1. What is your role in this?
   2. Where do you source research evidence from?
   3. Are there other stakeholders involved in the process of reviewing the research evidence?
      1. If yes, who are they?
   4. Do you collaborate or partner with other departments or organisations to receive support you in this process?
   5. Do you collaborate or partner with other departments or organisations to provide support you in this process?
   6. How is research evidence communicated to stakeholders?
   7. Does research evidence influence planning decisions?
      1. If yes, how?
      2. If no, why?
2. The Victorian Government recently mandated that LGAs must address tackling climate change and its impacts on health in 2021-2025 MPHWPs.
   1. Did you include this as a priority area in your 2021-2025 MPHWP?
      1. If yes, why?
      2. If no, why not?
   2. Was this a new focus area for your local government or were you already aware of and working to address the impacts of climate change on health in your local community?
   3. In 2020 the Victorian Government released "Guidance for local government" to provide guidelines for integrating tackling climate change and it's impacts on health in MPHWPs and highlight evidence about the impacts of climate change and strategies to both mitigate and respond to the health impacts of climate change. Did you draw on these guidelines?
      1. If yes, were they helpful? Why/Why not
         1. If no – why not? Did you draw on other guidelines?
      2. If yes - were they helpful? Why? (Specifics)
   4. What other research evidence did you draw on to develop strategies to address climate related public health concerns in your local area?
   5. Were there other stakeholders involved in this process?
      1. If yes, who were they?
   6. Did you collaborate of partner with other departments or organisations to receive support in this process?
      1. If yes, who? how?
   7. Did you collaborate of partner with other departments or organisations to provide support in this process?
      1. If yes, who? how?

**Abridged schedule for interviewees from sustainability/environmental teams**

The Victorian Government recently mandated that LGAs must address tackling climate change and its impacts on health in 2021-2025 MPHWPs.

1. Are public health outcomes considered in sustainability decision-making? Can you give a recent example of this?

2. Is research evidence considered an important resource? Why?

1. Was this a new focus area for your local government or were you already aware of and working to address the impacts of climate change on health in your local community?
2. In 2020 the Victorian Government released "Guidance for local government" to provide guidelines for integrating tackling climate change and it's impacts on health in MPHWPs and highlight evidence about the impacts of climate change and strategies to both mitigate and respond to the health impacts of climate change. Did you draw on these guidelines?
   1. If yes, were they helpful? Why/Why not
      1. If no – why not? Did you draw on other guidelines?
   2. If yes - were they helpful? Why? (Specifics)
3. What other research evidence did you draw on to develop strategies to address climate related public health concerns in your local area?
4. Were there other stakeholders involved in this process?
   1. If yes, who were they?
5. Did you collaborate of partner with other departments or organisations to receive support in this process?
   1. If yes, who? how?
6. Did you collaborate of partner with other departments or organisations to provide support in this process?
   1. If yes, who? how?
